# A cross-over, randomised feasibility study of digitally-printed versus hand-painted artificial eyes in adults (PERSONAL-EYE-S): health economic findings

**DOI:** 10.1186/s40814-025-01716-3

**Published:** 2025-11-03

**Authors:** Sarah Ronaldson, Elizabeth Coleman, Amie Woodward, Tim Zoltie, Paul Bartlett, Laura Wilson, Tom Archer, Jessica Kawalek, Florien Boele, Bernard Chang, George Kalantzis, Michael Theaker, Nabil El-Hindy, Emma Walshaw, Taras Gout, Judith Watson

**Affiliations:** 1 York Trials Unit, Department of Health Sciences, University of York, York, YO10 5DD UK; 2 School of Dentistry, Faculty of Medicine and Health, University of Leeds, Worsley Building, Leeds, LS2 9LU UK; 3 Leeds Artificial Eye Service, Leeds Dental Institute, Leeds Teaching Hospitals NHS Trust, Worsley Building, Leeds, LS2 9LU UK; 4 Leeds Institute of Medical Research at St James’s, St James’s University Hospital, University of Leeds, Leeds, LS9 7TF UK; 5 Leeds Institute of Health Sciences, Faculty of Medicine and Health, University of Leeds, Leeds, LS2 9JT UK; 6 Department of Ophthalmology, Leeds Teaching Hospitals NHS Trust, Leeds, LS9 7TF UK

**Keywords:** Economic evaluation, Cost effectiveness, Feasibility study, Randomised controlled trial, Artificial eyes

## Abstract

**Background:**

Technology advances mean alternatives to hand-painted artificial eyes are possible, but the feasibility of conducting a large-scale trial is unknown. The aim was to assess the feasibility of collecting healthcare resource use and associated costs needed to undertake a large-scale randomised controlled trial (RCT) comparing the effectiveness and cost-effectiveness of hand-painted artificial eyes with digitally-printed artificial eyes.

**Methods:**

Participants wore a digitally-printed artificial eye and a hand-painted artificial eye, for two weeks each, in a random order. Individual patient-level data was used to explore health outcomes (EQ-5D-5L) and resource use. Costs of the two artificial eye services were collected. A full economic evaluation was not conducted. An appropriate economic evaluation framework was developed to identify the relevant health economic data necessary for a future full trial.

**Results:**

Thirty-five participants were randomised. Response rates were 97–100% for the EQ-5D-5L. Resource use questions were less well completed: 54% complete responses at baseline, 40% partial responses and 6% missing/invalid responses. The two follow-up points had similar rates. Eye services cost data were well completed.

Mean utility was 0.77 after wearing the hand-painted eye and 0.83 after the digitally-printed eye. Average manufacturing times were 294 min (digitally-printed) and 355 min (hand-painted). Remake appointments were needed for digitally-printed eyes only. Estimated cost for the digitally-printed eye service is £404 and £347 for the hand-painted eye. Time between fitting and final evaluation was 56 days (digitally-printed eye) and 60 days (hand-painted). Results should be interpreted with caution, as estimates were based on a small sample.

**Conclusion:**

It was possible to collect EQ-5D-5L, healthcare resource use and manufacturing times allowing a costing to be calculated. Thus, a large-scale RCT with full cost-effectiveness analysis would feasible. Refinements are suggested. Future economic analysis should consider how best to evaluate the service, possibility with modelling rather than within-trial analysis only.

**Trial registration:**

ISRCTN85921622.

## Key messages regarding feasibility

‒ Lack of research in this area meant there were uncertainties in the ability to collect economic data.

‒ Standardised measures and clinician data were well completed; however, work is needed to refine resource use questionnaire.

‒ A full economic evaluation within a full-scale randomised controlled trial exploring the effectiveness of different artificial eyes is feasible.

## Background

It is estimated that within the United Kingdom there are 60,000 patients who have an artificial eye. [1]—with around 11,500 artificial eyes required to be manufactured each year, for new users, and to provide replacements for existing artificial eye users.

The current most used method by the National Artificial Eye Service (NAES), and globally, is to create a hand-painted artificial eye, where the artificial eye is colour-matched to the patient’s other eye. This should provide a life-like artificial eye, which may reduce anxiety and depression that patients with artificial eyes often experience [2–4]. However, the manufacturing of these can take 6 to 10 weeks and may require multiple revisions to ensure a good match—which may lead to distress for the patients.

There was an unmet patient need for a new manufacturing approach to create more realistic artificial eyes in a shorter period of time. The Leeds Artificial Eye Service (LAES) developed a novel digitally-printed artificial eye manufacture method and service provision model to create more life-like artificial eyes within two weeks.

The NIHR funded the PERSONAL-EYE-S study (A novel, high-definition PERSONALised artificial EYE Service) which was a cross-over, randomised controlled, open feasibility study. The primary objective was to assess whether it is feasible to conduct a future, full-scale randomised controlled trial (RCT) of the effectiveness and cost-effectiveness of digitally-printed artificial eyes compared to hand-painted eyes.

The embedded health economics component of the PERSONAL-EYE-S study investigated the feasibility of undertaking an economic evaluation of digitally-printed artificial eyes versus current standard care of hand-painted artificial eyes within a full-scale RCT. The trial was registered on the ISRCTN on 17th June 2021 (Registration number: ISRCTN85921622).

### Aims and objectives

The overall feasibility trial aimed to understand whether a full-scale trial would be feasible to undertake, by observing the number of eligible and consenting participants, as well as logistical implications of the trial. Additionally, we aimed to explore whether a health economic evaluation would be possible, using the following objectives:

- Explore the completion rate for the EQ-5D-5L [5].
- Explore the completion rate for the participant reported resource use.
- Explore the completion rate for clinical data, including time taken and staff grade for each element of the assessments.
- Explore the ability to collect and assign costs associated with the processes of both intervention arms.

We determined that for an outcome measure, if there was 10% or more missing data, modifications would be needed prior to use in the main trial. Completion rates for the various elements of the participant-reported resource use may identify worthwhile changes, optimise the completion of these and reduce burden on participants.

## Methods

A favourable ethical opinion was given by North West – Haydock and Health Research Authority approval obtained on 09th June 2021 (Reference 21/NW/0150). Full details of the study design are presented elsewhere [6]. In summary, potential participants were identified in clinic, via database screening, or via notification placed on the Royal National Institute of Blind People and Blind Veterans UK websites. Following an initial discussion, interested patients were checked for eligibility by LAES, and if appropriate, written consent was taken.

The target sample size of this feasibility trial was 35 participants, with an anticipated 15% attrition rate, allowing for approximately 30 participants in the analysis. This was deemed large enough to estimate within-subject standard deviations for outcomes measures and is in line with feasibility recommendations: further justification is included within the published protocol paper. [6]

The order in which the participant received and used the two eyes was randomised. Participants were randomised on a 1:1 basis to either:

- receive a digitally-printed artificial eye first, followed by a hand-painted artificial eye (‘digitally-printed first' group); or.
- receive a hand-painted artificial eye first, followed by a digitally-printed artificial eye (‘hand-painted first’ group).

Participants wore each eye for a period of up to two weeks each. They retained their original eye, so could choose to stop wearing the new eye at any point if they wished.

Participants attended up to five clinic appointments within the study:

i. Clinic one: eligibility and willingness to participate was assessed, randomisation and start manufacture of first eye;
ii. Clinic two: complete manufacture of first eye, and first eye provided;
iii. Clinic three: follow-up 1—follow-up data collected on the first eye, start of manufacture of second eye;
iv. Clinic four: complete manufacture of second eye;
v. Clinic five: follow-up 2—follow-up data collected on the second eye and qualitative interviews.

### Data sources

#### Health-related quality of life

Participants completed the EQ-5D-5L [5], at baseline and two follow-up points. The instrument measures health-related quality of life (HRQoL) in terms of five dimensions: mobility, self-care, usual activities, pain and discomfort, and anxiety and depression. For each dimension, participants rate the extent of their problems as ‘no problems’, ‘slight problems’, ‘moderate problems’, ‘severe problems’ or ‘extreme problems/unable’. A visual analogue scale (VAS) was also completed. A participant’s health status is defined by their five responses to the EQ-5D-5L descriptive system, with a single index utility assigned.

#### Healthcare resource use

Participants were asked about their use of healthcare services over the past 12 months (at baseline) and for the period since they were fitted with their most recent study eye (follow-up questionnaire). Data were obtained for resources used within primary care and the community (i.e., appointments with a GP, nurse and other primary/community care healthcare professionals) and the hospital setting (i.e., hospital outpatient attendances, inpatient admissions, day case attendances and accident and emergency admissions).

In addition, the time spent in the eye clinics as part of the eye services were collected and costed using data collected via a Clinic Record Booklet. Specifically, the booklet was used to record the time spent on photographing the eyes, and on manufacturing of the eyes, which comprised a list of individual items such as review by maxillofacial technician, photographic printing, hand paint, insert corneal unit, deflask, trim and polish. Clinicians marked against these items as to whether the task had been completed or not at the specific clinic (i.e., clinic 1 to 5), with the corresponding time taken and clinician who undertook the task. There was also space on the form to add further tasks that were not included in the pre-specified list.

#### Analyses

The economic analysis involved developing an appropriate economic evaluation framework, identifying the relevant health economic data to be collected in the study, and considering the feasibility of data collection methods in order to inform a future full trial. The individual patient-level data collected as part of the feasibility study was used to explore health outcomes (i.e., the EQ-5D-5L), resource use and costs of the two artificial eye services. Analyses were undertaken using STATA version 17 [7].

The EQ-5D-5L was scored according to the EuroQol User Guide [8], following the mapping function developed by van Hout et al. (2012) [9]. Completeness of the EQ-5D-5L data was examined, with the proportion of missingness reported by study arm. Descriptive statistics of the utility scores were presented at the three data collection points.

The primary care, community and hospital setting data were used to consider the appropriateness of the resource use questionnaire, to refine the questionnaire where required for potential future use. For example, where items repeatedly appear in ‘other’ boxes (i.e., for other outpatient appointments and other primary/community care appointments), we would consider adding these as separate resource use items for questionnaires used in a future full-scale trial.

Due to the work being part of a feasibility study, a full economic evaluation was not conducted. The findings from the health economics component will be used to improve and refine the methods that would be used for an economic evaluation in a full-scale trial. This could include the improvement of completion rates for health economic data and ensuring accurate representation of the patient pathway by considering the relevant costs and outcomes associated with digitally-printed or hand-painted artificial eyes for patients requiring an artificial eye.

#### Costs

A National Health Service (NHS) perspective was taken for the costing, with data regarding healthcare utilisation collected at the three time points to capture relevant resource use for patients with an artificial eye. Information collected on such items was used to inform the design of resource use questions in a full-scale trial. Resource use data were examined for completeness, in terms of the proportion of missing data, to help guide which items would be collected for a full economic evaluation in a full-scale trial. Descriptive statistics were presented for resource use by randomised group (i.e., mean, median, standard deviation, minimum, maximum). Unit costs are presented to provide indicative costs for a full economic evaluation.

The costs of the two different eye services were estimated to indicate the likely resource implications. For instance, the number, duration, and times between clinics were incorporated, using data from the study to estimate the costs of the eye services. Unit costs of the time spent by relevant clinicians involved in the clinics were applied according to the individual tasks completed at the clinics, collected using a Clinic Record Booklet completed by clinicians. The materials of the eyes and the equipment were assumed to be the same, with the focus of this costing instead being on the manufacturing and clinic times.

Unit costs for the resources used were obtained from established costing sources such as NHS Reference Costs [10] and PSSRU Unit Costs of Health and Social Care [11] (Table 1). All costs were evaluated in 2020–21 pounds sterling (£). In cases where costs were sourced from previously published data, costs were inflated to 2020–21 prices. The staff costs that fed into the intervention cost comprised band 5 (£41 per hour), band 6 (£54 per hour) and band 7 (£65 per hour) [11].

**Table 1:** Completion rates for the EQ-5D-5L and resource use

	**Hand-painted first**^**a **^**(*n* = 18)**			**Digitally-printed first**^**b **^**(*n* = 17)**		
Measure	**Complete**	**Partially complete**	**Missing/invalid response**	**Completed**	**Partially complete**	**Missing/invalid response**
EQ-5D-5L						
Baseline (n = 35)	18 (100%)	0 (0%)	0 (0%)	16 (94%)	1 (6%)	0 (0%)
Follow-up 1 (n = 30)	16 (100%)	0 (0%)	0 (0%)	13 (93%)	1 (7%)	0 (0%)
Follow-up 2 (n = 30)	15 (100%)	0 (0%)	0 (0%)	15 (100%)	0 (0%)	0 (0%)
EQ-5D-5L VAS						
Baseline (n = 35)	18 (100%)	0 (0%)	0 (0%)	17 (100%)	0 (0%)	0 (0%)
Follow-up 1 (n = 30)	16 (100%)	0 (0%)	0 (0%)	14 (100%)	0 (0%)	0 (0%)
Follow-up 2 (n = 30)	15 (100%)	0 (0%)	0 (0%)	14 (93%)	0 (0%)	1 (7%)
Resource use						
Baseline (n = 35)	9 (50%)	8 (44%)	1 (6%)	10 (59%)	6 (35%)	1 (6%)
Follow-up 1 (n = 30)	9 (56%)	6 (38%)	1 (6%)	9 (64%)	5 (36%)	0 (0%)
Follow-up 2 (n = 30)	9 (60%)	6 (40%)	0 (0%)	8 (53%)	7 (47%)	0 (0%)

^a^ for the hand-painted first group, follow-up 1 shows resource use/EQ-5D data whilst fitted with a hand-painted eye, and follow-up 2 shows resource use/EQ-5D data whilst fitted with a digitally-printed eye;

^b^ for the digitally-printed first group, follow-up 1 shows resource use/EQ-5D data whilst fitted with a digitally-printed eye, and follow-up 2 shows resource use/EQ-5D data whilst fitted with a hand-painted eye

Note: response rates are presented as a proportion of the number of participants who responded at each time point, e.g., of 30 participants at follow-up 1

## Results

### Participants

Thirty-five participants were randomised to the feasibility study, with a mean age of 52.8 years (SD 17.2; range 21 to 89). Twenty-one (60%) were male and the majority (82.9%) were of a white British ethnicity.

### Health related quality of life (EQ-5D-5L)

#### Completeness of data response

Response rates were high across the three time points for the participant self-completed EQ-5D-5L questionnaires. Of the 35 participants at baseline, 34 (97%) completed the EQ-5D-5L descriptive system fully and one (3%) completed it partially. At follow-up 1, of the 30 who responded, 29 (97%) completed the EQ-5D-5L in full, with one (3%) partial completion. At follow-up 2, all of the 30 responders provided complete EQ-5D-5L responses.

#### EQ-5D-5L quality of life scores

Participants’ EQ-5D-5L responses showed baseline utility scores to be similar for the two groups, at 0.74 on average. Mean utility increased to 0.77 after wearing the hand-painted eye, whilst mean utility increased to 0.83 after the digitally-printed eye had been worn. Full details are in Tables 1 and 2.

**Table 2:** Number of missing dimensions for invalid EQ-5D-5L questionnaires

EQ-5D-5L	Hand-painted first (*n* = 18) Number of missing dimensions					Digitally-printed first (*n* = 17) Number of missing dimensions				
Follow up	**1**	**2**	**3**	**4**	**5**	**1**	**2**	**3**	**4**	**5**
Baseline	0	0	0	0	0	1	0	0	0	0
Follow-up 1	0	0	0	0	0	1	0	0	0	0
Follow-up 2	0	0	0	0	0	0	0	0	0	0

### Resource use and healthcare costs

#### Completion of data response

The resource use questions were not completed as comprehensively as the EQ-5D-5L: at baseline, 19 (54%) provided complete responses to all resource use questions, 14 (40%) provided partial responses and two (6%) had a missing/invalid response. Response rates remained similar at the two follow-up points, and response to the individual items ranged between 0 and 94%.

#### Resource use frequency

The healthcare services used by this population appeared to be captured by the resource use items included in the questionnaire; however, some items had low/no usage reported and hence would likely be removed in a full trial. For instance, participants reported zero appointments with both the optometrist and orthoptist at all time points, hence these could be items to be considered for removal; they could still be picked up by inclusion of a free-text ‘other’ item in the questionnaire however. See Table 3 for details.

**Table 3:** Mean resource use

	**Hand-painted first (*n* = 18)**			**Digitally-printed first (*n* = 17)**		
	**n**	**Mean (SD)**	**Median (Min, Max)**	**n**	**Mean (SD)**	**Median (Min, Max)**
GP appointments at GP practice						
Baseline	17	0.24 (0.56)	0 (0, 2)	16	0.13 (0.34)	0 (0, 1)
Follow-up 1	15	0 (0)	0 (0, 0)	13	0 (0)	0 (0, 0)
Follow-up 2	14	0 (0)	0 (0, 0)	14	0.07 (0.27)	0 (0, 1)
GP appointments over the phone/online						
Baseline	17	0.12 (0.33)	0 (0, 1)	16	0 (0)	0 (0, 0)
Follow-up 1	15	0 (0)	0 (0, 0)	13	0.15 (0.38)	0 (0, 1)
Follow-up 2	14	0 (0)	0 (0, 0)	13	0 (0)	0 (0, 0)
Mental health services (face-to-face)						
Baseline	17	0 (0)	0 (0, 0)	16	0 (0)	0 (0, 0)
Follow-up 1	15	0 (0)	0 (0, 0)	13	0 (0)	0 (0, 0)
Follow-up 2	14	0 (0)	0 (0, 0)	13	0.15 (0.55)	0 (0, 0)
Mental health services (phone/online)						
Baseline	17	0 (0)	0 (0, 0)	16	0.25 (1.00)	0 (0, 4)
Follow-up 1	15	0 (0)	0 (0, 0)	13	0 (0)	0 (0, 0)
vFollow-up 2	14	0 (0)	0 (0, 0)	13	0 (0)	0 (0, 0)
Occupational therapist appointments						
Baseline	16	0 (0)	0 (0, 0)	15	0.07 (0.26)	0 (0, 1)
Follow-up 1	15	0.07 (0.26)	0 (0, 1)	13	0 (0)	0 (0, 0)
Follow-up 2	13	0 (0)	0 (0, 0)	13	0 (0)	0 (0, 0)
Other primary/community care appointments						
Baseline	13	0.38 (0.96)	0 (0, 3)	12	0.25 (0.87)	0 (0, 3)
Follow-up 1	13	0 (0)	0 (0, 0)	13	0 (0)	0 (0, 0)
Follow-up 2	13	0 (0)	0 (0, 0)	11	0 (0)	0 (0, 0)
Maxillofacial outpatient clinic visits						
Baseline	17	0.06 (0.24)	0 (0, 1)	15	0 (0)	0 (0, 0)
Follow-up 1	14	0 (0)	0 (0, 0)	13	0 (0)	0 (0, 0)
Follow-up 2	14	0 (0)	0 (0, 0)	15	0 (0)	0 (0, 0)
Ophthalmology/eye outpatient clinic visits						
Baseline	17	0.18 (0.53)	0 (0, 2)	15	0.07 (0.26)	0 (0, 1)
Follow-up 1	14	0 (0)	0 (0, 0)	13	0 (0)	0 (0, 0)
Follow-up 2	14	0 (0)	0 (0, 0)	14	0 (0)	0 (0, 0)
Optometrist outpatient clinic visits						
Baseline	17	0 (0)	0 (0, 0)	15	0 (0)	0 (0, 0)
Follow-up 1	14	0 (0)	0 (0, 0)	13	0 (0)	0 (0, 0)
Follow-up 2	14	0 (0)	0 (0, 0)	15	0 (0)	0 (0, 0)
Orthoptist outpatient clinic visits						
Baseline	17	0 (0)	0 (0, 0)	15	0 (0)	0 (0, 0)
Follow-up 1	14	0 (0)	0 (0, 0)	13	0 (0)	0 (0, 0)
Follow-up 2	14	0 (0)	0 (0, 0)	15	0 (0)	0 (0, 0)
National Artificial Eye Service clinic visits						
Baseline	17	0.59 (0.94)	0 (0, 3)	16	0.63 (1.09)	0 (0, 3)
Follow-up 1	14	0.14 (0.53)	0 (0, 2)	13	0 (0)	0 (0, 0)
Follow-up 2	14	0 (0)	0 (0, 0)	15	0.07 (0.26)	0 (0, 1)
Radiology outpatient appointments						
Baseline	17	0 (0)	0 (0, 0)	15	0.13 (0.52)	0 (0, 2)
Follow-up 1	14	0.5 (1.61)	0 (0, 6)	13	0 (0)	0 (0, 0)
Follow-up 2	14	0 (0)	0 (0, 0)	15	0.13 (0.35)	0 (0, 0)
Other outpatient appointments (face-to-face)						
Baseline	14	0.14 (0.53)	0 (0, 2)	10	0 (0)	0 (0, 0)
Follow-up 1	11	0 (0)	0 (0, 0)	11	0 (0)	0 (0, 0)
Follow-up 2	12	0 (0)	0 (0, 0)	12	0 (0)	0 (0, 0)
Other outpatient appointments (phone/online)						
Baseline	12	0.17 (0.58)	0 (0, 2)	8	0 (0)	0 (0, 0)
Follow-up 1	12	0 (0)	0 (0, 0)	11	0 (0)	0 (0, 0)
Follow-up 2	11	0 (0)	0 (0, 0)	11	0 (0)	0 (0, 0)
A&E attendances						
Baseline	17	0 (0)	0 (0, 0)	15	0 (0)	0 (0, 0)
Follow-up 1	15	0.27 (0.80)	0 (0, 3)	14	0 (0)	0 (0, 0)
Follow-up 2	15	0 (0)	0 (0, 0)	14	0 (0)	0 (0, 0)
Day case hospital attendances						
Baseline	17	0.18 (0.39)	0 (0, 1)	14	0 (0)	0 (0, 0)
Follow-up 1	15	0 (0)	0 (0, 0)	14	0 (0)	0 (0, 0)
Follow-up 2	15	0 (0)	0 (0, 0)	14	0 (0)	0 (0, 0)
Inpatient nights in hospital						
Baseline	17	0 (0)	0 (0, 0)	15	0 (0)	0 (0, 0)
Follow-up 1	15	0 (0)	0 (0, 0)	14	0 (0)	0 (0, 0)
Follow-up 2	15	0 (0)	0 (0, 0)	14	0 (0)	0 (0, 0)

Unless otherwise stated, appointments reported in the table were face-to-face

Note: There were zero appointments reported for the following outpatient appointments that took place over the phone/online, hence they were not included in the table: Maxillofacial, Ophthalmology/eye, Optometrist, Orthoptist, NAES, radiology outpatient clinic appointments (over the phone/online)

The most commonly used healthcare services reported by participants were: GP appointments (at the GP practice and over the phone/online), NAES appointments, radiology, ophthalmology outpatient, and ‘other’ appointments (both primary/community and hospital outpatients). The majority of resource use items that asked participants about phone/online appointments had zero uptake recorded against them, hence these could potentially be removed for a future trial.

#### Costs

The Clinic Record Booklet, used to estimate the cost of the eye services, was well completed, with only low levels of missing data. A high level of detail was recorded on the forms; however, this could be revisited if a full trial were to go ahead to determine whether efficiencies could be made in terms of staff time spent on completing the form.

The mean manufacturing times were 294.3 min (SD 69.9) for digitally-printed eyes and 354.6 min (SD 34.1) for hand-painted eyes; hence, manufacturing times for digitally-printed eyes were on average 60 min shorter. However, there were nine additional appointments for re-makes needed for digitally-printed eyes versus none for the hand-painted eyes.

The cost of the digitally-printed eye service was estimated to be £403.96 (SD £135.43) and the hand-painted eye service was estimated at £346.82 (SD £33.30), based on the time taken at the clinics attended and incorporating re-make time – a difference of £57.14. In addition to the costs provided, there were nine booked appointments that were not attended, which would have incurred a cost due to wasted clinic/staff time. If the cost of £160 of a missed appointment [12] were attached to this, it would equate to a total of £1,440 wasted on missed appointments.

The interval between clinics, i.e., between fitting and final evaluation of the eye, was similar for the two eye services: mean 60.2 (SD 43.1) days for hand-painted and 56.4 (SD 38.4) days for digitally-printed. Participants reported that they visited a prosthetist for a clean and polish every 16.6 months on average (SD 18.4). In some instances, clinics 3 and 4 were combined, which both reduces participant burden, and associated staff time – so may worth considering in a future RCT.

Unit costs for the resources that would feed into a full economic evaluation were largely available from published sources, with some supplemented by information from the study team (Table 4). The specific member of staff who undertook each task at the clinics was recorded and costed accordingly. A sensitivity analysis was conducted to explore the impact of instead applying an average cost of the relevant staff bands for each task that had more than one staff band involved in undertaking them. The hand-painted group still had lower costs associated with them, on average, though the difference in cost between the two groups reduced overall; £382.85 (£133.17) for digitally-printed versus £369.04 (£34.84) for hand-painted, a difference of £13.81.

**Table 4:** Unit costs of healthcare services

Appointment/attendance	Unit of measurement	Unit cost	Notes	Source
GP appointment at GP practice	Per appointment	£39.23	Per consultation of 9.22 min duration (including direct care staff costs and qualifications)	PSSRU 2021
GP appointment over the phone/online	Per appointment	£30.53	Per phone contact of 7.1 min duration (PSSRU 2015); at £4.30 per minute of patient contact (PSSRU 2021)	PSSRU 2021 & 2015
Mental health support services	Per appointment	£132.00	IAPT contact, p24, NHS national costing data for mental health services	PSSRU 2021
Occupational therapist appointment	Per appointment	£50.00	Cost per hour (including training) of community occupational therapist (local authority)	PSSRU 2021 (p125)
Maxillofacial outpatient clinic	Per attendance	£197.91	Maxillo-Facial Surgery, Total Outpatient Attendance sheet	NHS Ref Costs 20/21
Ophthalmology/eye outpatient clinic	Per attendance	£168.24	Ophthalmology, Total Outpatient Attendance sheet	NHS Ref Costs 20/21
Optometrist outpatient clinic	Per attendance	£135.48	Optometry, Total Outpatient Attendance sheet	NHS Ref Costs 20/21
Orthoptist outpatient clinic	Per attendance	£131.17	Orthoptics, Total Outpatient Attendance sheet	NHS Ref Costs 20/21
National Artificial Eye Service clinic	Per attendance	£168.24	Assume same as Ophthalmology/eye outpatient clinic cost	NHS Ref Costs 20/21
Radiology outpatient appointment	Per attendance	£139.13	Average of: plain film x-ray £57.78 (PF), CT-scan £148.38 (RD20A) and MRI scan £211.24 (RD01A) – Diagnostic Imaging Sheet	NHS Ref Costs 20/21
Accident and Emergency department attendance	Per attendance	£296.87	Per (non-specific) attendance	NHS Ref Costs 20/21
Day case hospital attendance	Per attendance	£1191.96	Per (non-specific) attendance	NHS Ref Costs 20/21
Inpatient night in hospital	Per inpatient night	£372.70	Total expenditure on excess bed days (elective and non-elective) divided by total activity, inflated to 2021 prices	NHS Ref Costs 17/18
Check and polish with prosthetist	Per appointment	£27.00	30 min of prosthetist time at £54 (for band 6) per hour	PSSRU Unit Costs & Study Team

Note that ‘other’ primary/community care appointments and ‘other’ outpatient appointments were costed according to the responses provided by participants; Unless specifically stated otherwise, costs were assumed to be the same for face-to-face and phone/online appointments

## Discussion

This feasibility study has estimated the costs of two different artificial eye services and has explored whether a full trial into the cost-effectiveness of digitally-printed versus hand-painted artificial eyes would be feasible. It has been established that a future large-scale RCT would be able to undertake a full cost-effectiveness analysis providing refinements to trial design and documentation are made.

Data relating to EQ-5D-5L, healthcare resource use, and manufacturing times for the artificial eyes, were found to be collectable, allowing for a costing to be calculated. However, undertaking this early health economics work, lessons have been learned as to how to design participant-facing and clinician-completed CRFs in order to target information required for a full economic evaluation.

The additional appointments required for re-makes for the digitally-printed eye meant that additional clinic time was needed, hence additional costs attached to the staff time involved. Although the manufacturing times were reduced for digitally-printed eyes, the extra re-makes resulted in costs being higher overall for the digitally-printed eyes group.

The Clinic Record Booklet gathered detailed information on the tasks that were undertaken for each participant at the clinic visits. The responses provided in the study here indicate that for some of the tasks that were conducted, a similar time was taken for these, for instance the time spent for photographing the eye, or time spent for maxillofacial technician (or equivalent) review. Such items could be approximated in a full trial by use of a sample for instance, rather than collecting time data for all participants, which would potentially streamline the data collection forms for clinicians. However, for other items, such as the time spent hand painting, there was more variation in the responses, plus with this particular item being a key driver, it would be more important to capture for each participant.

Low resource use was reported by participants; this was expected for the follow-up questionnaires due to the short periods they asked about (i.e., only a few weeks). However, the resource use collected at baseline, for the last 12 months, was also relatively low overall. The resources with highest use were GP appointments, ophthalmology outpatient appointments, NAES appointments, radiology appointments and ‘other’ appointments (both primary/community and hospital outpatients). A future economic analysis should consider how best to evaluate the service, with the possibility of modelling rather than within-trial analysis only.

The health economic component of the study found that it was feasible to collect the data needed for a future full economic evaluation to be undertaken as part of a randomised controlled trial. High response levels were recorded for the EQ-5D-5L at all time points, and similarly for the clinician-reported data used to feed into the eye service cost estimates, indicating potential replication in a larger trial. Some updates could be made to the resource use questionnaire and possibly to the clinician-completed forms based on information gained from this study. The cost of the two eye services was estimated – £404 for digitally-printed and £347 for hand-painted – with the finding that manufacturing times were shorter for digitally-printed eyes, but more re-makes were required. However, it is important to remember that these estimates were based on a population of 35 participants recruited to the study. A full economic evaluation conducted as part of a full-scale randomised controlled trial would be able to comprehensively investigate the cost-effectiveness of the two eye services. Implementation of the cost-effective eye service has the potential to bring about benefits to patients and cost savings, thereby providing the most efficient service for patients.

## Conclusions

A full economic evaluation will be feasible to undertake in a full-scale randomised controlled trial and would allow for a thorough investigation of the cost-effectiveness of the hand-painted versus digitally printed artificial eyes. Standardised measures such as the EQ-5D were well completed, and are suitable for use in a full evaluation, however further thought is required into refining questionnaires on service use and clinical appointments to minimise missing data.

## Data Availability

The data that support the findings of this study are available from the corresponding author, upon reasonable request.

## Abbreviations

CRF: Case Report Form
HRQoL: Health-related quality of life
LAES: Leeds Artificial Eye Service
NAES: National Artificial Eye Service
NHS: National Health Service
RCT: Randomised controlled trial
VAS: Visual analogue scale

## References

[1] Viswanathan P, Sagoo MS, Olver JM. UK national survey of enucleation, evisceration and orbital implant trends. Br J Ophthalmol. 2007;91(5):616–9. 17151061 10.1136/bjo.2006.103937 PMC1954760

[2] Song JS, Oh J, Baek SH. A survey of satisfaction in anophthalmic patients wearing ocular prosthesis. Graefes Arch Clin Exp Ophthalmol. 2006;244(3):330–5. 16133031 10.1007/s00417-005-0037-0

[3] Lubkin V, Sloan S. Enucleation and psychic trauma. Adv Ophthalmic Plast Reconstr Surg. 1990;8:259–62. 2248718

[4] McBain HB, et al. The psychosocial impact of living with an ocular prosthesis. Orbit. 2014;33(1):39–44. 24205995 10.3109/01676830.2013.851251

[5] Hardman M, et al. Development and preliminary testing of the new five-level version of EQ-5D (EQ-5D-5L). Qual Life Res. 2011;20(10):1727–36. 21479777 10.1007/s11136-011-9903-x PMC3220807

[6] Gout T, Zoltie T, Woodward A, et al. A cross-over, randomised feasibility study of digitally printed versus hand-painted artificial eyes in adults: PERSONAL-EYE-S - a study protocol [version 2; peer review: 2 approved]. NIHR Open Res. 2023;2:50. 37056714 10.3310/nihropenres.13311.2 PMC7614426

[7] StataCorp, Stata Statistical Software: Release 17. 2021, StataCorp LLC: College Station, TX.

[8] EuroQol Research Foundation, EQ-5D-5L User Guide: Basic information on how to use the EQ-5D-5L instrument. Available from: https://euroqol.org/publications/user-guides 2019.

[9] van Hout B, et al. Interim scoring for the EQ-5D-5L: mapping the EQ-5D-5L to EQ-5D-3L value sets. Value Health. 2012;15(5):708–15. 22867780 10.1016/j.jval.2012.02.008

[10] Jones K, Burns A. Unit costs of health and social care 2021. Canterbury: Personal Social Services Research Unit: University of Kent; 2021.

[11] Curtis, L. and A. Burns, Unit Costs of Health and Social Care 2020. 2020, Personal Social Services Resaerh UnitL Universiry of Kent, Canterbury.

[12] https://www.bi.team/blogs/reducing-missed-appointments/ [Last accessed 16 June 2023]

